# In Vitro Effects of PTH (1-84) on Human Skeletal Muscle-Derived Satellite Cells

**DOI:** 10.3390/biomedicines11041017

**Published:** 2023-03-27

**Authors:** Cecilia Romagnoli, Roberto Zonefrati, Elena Lucattelli, Marco Innocenti, Roberto Civinini, Teresa Iantomasi, Maria Luisa Brandi

**Affiliations:** 1 Department of Experimental and Clinical Biomedical Sciences, University of Florence, 50139 Florence, Italy; cecilia.romagnoli@unifi.it (C.R.);; 2 Italian Foundation for the Research on Bone Diseases (F.I.R.M.O.), 50129 Florence, Italy; 3 Plastic and Reconstructive Microsurgery, Careggi University Hospital, 50134 Florence, Italy; 4 IRCCS Istituto Ortopedico Rizzoli, 40136 Bologna, Italy; 5 Orthopedic Unit, Department of Health Sciences, University of Florence, C.T.O., 50139 Florence, Italy

**Keywords:** PTH (1-84), parathormone, skeletal muscle cells, myogenesis, myotube, hypoparathyroidism

## Abstract

Parathyroid hormone (PTH) is a hormone secreted by the parathyroid glands. Despite its well-known characterized anabolic and catabolic actions on the skeleton, the in vitro effects of PTH on skeletal muscle cells are limited and generally performed on animal models. The aim of this study was to evaluate the effects of a short impulse of PTH (1-84) on the proliferation and the differentiation of skeletal muscle satellite cells isolated from human biopsies. The cells were exposed for 30 min to different concentrations of PTH (1-84), from 10^−6^ mol/L to 10^−12^ mol/L. ELISA was used to assay cAMP and the myosin heavy-chain (MHC) protein. The proliferation was assayed by BrdU and the differentiation by RealTime-qPCR. A statistical analysis was performed by ANOVA followed by Bonferroni’s test. No significant variations in cAMP and the proliferation were detected in the isolated cells treated with PTH. On the other hand, 10^−7^ mol/L PTH on differentiated myotubes has shown significant increases in cAMP (*p* ≤ 0.05), in the expression of myogenic differentiation genes (*p* ≤ 0.001), and in the MHC protein (*p* ≤ 0.01) vs. untreated controls. This work demonstrates for the first time the in vitro effects of PTH (1-84) on human skeletal muscle cells and it opens new fields of investigation in muscle pathophysiology.

## 1. Introduction

Parathyroid hormone or parathormone (PTH) is a single-chain polypeptide of 84 amino acids, synthetized and secreted by the parathyroid glands, which are located posterior to the thyroid gland in the neck. The molecule is initially released as a precursor peptide of 115 amino acids and progressively cleaved, leaving 84 amino acids of the active form that are stored in granules and secreted in a circadian and pulsatile fashion or intracellularly degraded [1].

The main role of PTH is to control calcium homeostasis by increasing serum levels, regulating calcium absorption in the small intestine (indirectly, due to the action of calcitriol), renal reabsorption, and removal from the bone matrix [2]. PTH also inhibits the reabsorption of phosphate by the kidney tubules, thereby decreasing serum phosphate concentrations [3]. Therefore, PTH is the chief mediator of calcium and phosphate metabolism and carries out its action through interactions with receptors in two principal target organs, specifically bone and the kidney.

The classical actions of PTH (1-84) are mainly mediated through the PTH type 1 receptor (PTH1R), a G-protein-coupled receptor (GPCR) expressed on the surface of osteoblasts and osteocytes in bone and tubular cells in the kidney and are coupled to the production of second messenger adenosine 3′,5′-monophosphate (cyclic AMP, or cAMP) [4]. In fact, the activation of GPCRs results in the activation of heterodimeric G proteins, including those containing stimulatory subunit Gα_s_ which in turn stimulates membrane-bound adenylyl cyclase (AC), which converts adenosine triphosphate to cAMP [5]. In humans, PTH activates a second PTH receptor, called PTH2R, for which the tissue distribution differs significantly from the PTH1R [6]. The PTH2R is abundantly expressed in the central nervous system (hypothalamus) but also in the pancreas, testes, and placenta, as well as the vascular pole of the glomerulus, and the role for the PTH2R is still being defined [7,8]. The stimulation of the PTH2R by PTH also increases the cellular cAMP [6]. However, PTH has a much higher affinity for the PTH1R compared with the PTH2R and stimulates the PTH1R at much lower concentrations [9].

Several studies have demonstrated that PTH affects the cell functions of many organs that are not traditional targets of the hormone. The mRNAs of PTH receptors were found widely distributed in other tissues, such as the heart, brain, liver, pancreas, and others, where the acute exposure of PTH results in an increase in the second messenger cAMP levels, suggesting that these organs also contain PTH receptors and that their response to PTH involves an interaction between this hormone and its receptors [10].

PTH is recognized to be an important modulator of bone metabolism through its effects on osteoblasts and osteoclasts and the relative anabolic (bone formation) or catabolic (bone resorption) actions, depending on the duration and periodicity of the exposure [11,12]. Because bone and muscle interact anatomically and biochemically, they are considered a single functional compartment. While PTH is recognized as an important modulator of bone metabolism, the effects of the hormone on the skeletal muscle are poorly understood.

The hypothesis that PTH may affect skeletal muscle is supported by the fact that mRNA for both PTH receptors is expressed in this tissue, providing the molecular basis for recognition of the skeletal muscle as a target tissue of PTH [8,10,13,14]. Moreover, it was found that intact PTH affects muscle protein and amino-acid metabolisms, enhancing muscle proteolysis and increasing the release of alanine and glutamine, leading to an alteration in the amino-acid metabolism [15]. It was demonstrated that PTH affects the bioenergetics of skeletal muscle, inhibiting energy production, transfer, and utilization [16]. Moreover, it has been clinically reported that an excess of PTH, such as in primary hyperparathyroidism, is characterized by muscle involvement, whereas in chronic deficiency or an absence of PTH, hypoparathyroidism skeletal muscle symptoms, such as cramps and seizures, are well-recognized complications [17]. Finally, it has been reported that PTH, when used to treat adult patients with hypoparathyroidism, induces an improvement in mental and physical performance [18,19].

Because the biologically active part of the full-length PTH is represented by the first 34 amino acids of the N-terminal peptide sequence [17], the majority of the in vivo/in vitro works in the literature take into consideration the effect of the 1-34 fragment of PTH on different models.

Recently, an article by Sato et al. reported the in vivo effects of human recombinant PTH (1-34) on bone and skeletal muscle in a rat model of osteoporosis and muscle atrophy. Their data showed that monotherapy with PTH (1-34), at a dosage of 30 µg/kg body weight 3 times per week, significantly increased the proximal and distal femoral bone mineral density (BMD) and the percentage of the skeletal muscle mass in ovariectomized, tail-suspended rats [20]. Fujimaki et al. investigated the effect of PTH (1-34) on skeletal muscle and dysfunction using a murine ovariectomized model; mice that received intraperitoneal injections of 80 µg/kg body weight 3 times a week for 20 weeks significantly ameliorated muscle weakness and increased the fiber cross-sectional area and satellite cells activity, but not the muscle weight [21].

An article reported that PTH (1-34) and growth hormone prevented disuse osteopenia and sarcopenia in rats [22], and in another study, PTH (1-34) treatment significantly improved muscle weakness in a dystrophin-deficient mdx mouse model [23]. Moreover, a report showing that PTHR1 expression is required for myocyte differentiation, and that PTH (1-34) accelerated myogenesis and the production of myotubes in a mouse cellular model, highlights the importance of PTH in skeletal muscle regeneration [24]. Recently, our research group developed an in vitro model of human skeletal muscle cells isolated from biopsies, and we have shown an increase in *PTHR1* expression during myogenesis, supporting the possible role of PTH in skeletal muscle regeneration and its function [25].

Although the effects of PTH remain unknown in humans, the possibility of the hormone to ameliorate muscle atrophy and muscle weakness is foreseeable. Therefore, in an effort to gain new insight about the direct function of PTH on human skeletal muscles and considering the short half-life of the hormone in the circulation, the aim of the present study was to investigate the in vitro effects of a 30-min exposure to PTH (1-84), which is the native hormone, on the proliferation and the myogenesis of skeletal muscle satellite cells isolated from human biopsies (hSCs).

## 2. Materials and Methods

### 2.1. Primary Cultures of hSCs, Amplification, and Myogenic Differentiation

Human skeletal muscle biopsies were obtained from the discarded tissues of vastus medialis, pectoralis major, and rectus abdominis muscles of three healthy female volunteers of ages of 29, 36, and 52, respectively, undergoing plastic surgery, after signing an informed consent in accordance with a protocol approved by the Local Ethics Committee of the University Hospital Careggi (AOUC), Florence (Italy), for human studies (Ref. 19471_BIO), as well as the ethical standards stated in the Declaration of Helsinki (1964) and its later amendments, or comparable ethical standards.

Biopsies were cut in two pieces: one part was stored in RNAlater^®^ (Sigma-Aldrich, St. Louis, MO, USA, R0901) for molecular experiments on the tissue, and the second one was processed to obtained primary cells. The primary cells were characterized and amplified in matrigel^®^-coated plates (BD Company, Franklin Lakes, NJ, USA, 354234), in order to increase cell adherence and maintain cell phenotype, using Skeletal Muscle Cell Growth Medium (PromoCell GmbH, Heidelberg, Germany) and differentiated in myotubes using Skeletal Muscle Cell Differentiation Medium (PromoCell GmbH, Heidelberg, Germany), as previously described [25].

### 2.2. Immunofluorescence Staining of Cells

The immunofluorescence staining of cells was performed after their myogenic differentiation to myotubes in order to evaluate the presence of one of the main terminal myogenic differentiation markers, the myosin heavy-chain (MHC) protein, as previously described [26]. Samples were observed with laser scanning confocal microscopy (LSCM), using an LSM 510 Meta microscope equipped with ZEN 2009 Software (Carl Zeiss, Oberkochen, Germany).

### 2.3. PTH (1-84) Treatment

Different concentrations of PTH (1-84) (Sigma-Aldrich, St. Louis, MO, USA, P7036) from 10^−6^ to 10^−12^ mol/L were used in the experiments with proliferating hSCs or differentiated myotubes, with stimulation times of 30 min each.

### 2.4. RNA Extraction and PCR Analysis

RNA was isolated from the cellular pellets using QIAzol Lysis Reagent (Qiagen, Hilden, Germany, 79306) and 500 ng of the isolated RNA was reverse transcribed using the QuantiTect Reverse Transcription kit (Qiagen, Hilden, Germany, 205310). *Actin Beta* (*ACTB*) housekeeping gene was used as an internal control for sample transcription in order to verify that all samples were transcribed in a good way, starting from RNA. All the obtained amplicons were verified by sequencing analysis using BigDye™ Terminator v1.1 Cycle Sequencing Kit based on Sanger sequencing reactions (Thermo Fisher Scientific, Waltham, MA, USA, 4337451).

Qualitative PCR (q-PCR) was performed to evaluate the expression of the *PTH1R* and *PTH2R* genes in human skeletal muscle tissues, in hSCs, and in differentiated myotubes.

Real-Time Quantitative PCRs (RealTime-qPCR) were performed in human skeletal muscle tissues, in proliferating hSCs, and in differentiated myotubes, for *PTH1R* and *PTH2R* analysis, whereas for myoblast determination protein 1 (*MYOD1*), *MYOGENIN*, and myosin heavy-chain 2 (*MYH2)* gene analysis was performed in proliferating hSCs and after 6 and 24 h of myogenic differentiation, treated or not for 30 min with PTH (1-84) at the concentration of 10^−7^ mol/L. RealTime-qPCR reactions were carried out with TaqMan 5′-exonuclease assays, using a Rotor-Gene^®^Q Thermocycler (Qiagen, Hilden, Germany, 205310). The cDNA samples used for the construction of standard curves for quantitative analysis were subjected to PCR amplification for each gene. The standard curves were generated by assessing serial cDNA dilutions (10-fold dilution for 8 logarithms). Negative control tubes with water were included in each real-time PCR run to detect any carry-over contamination. Target gene expression was normalized to ribosomal protein S18 (*RPS18*) housekeeping gene and data were expressed as means ± SD of the number of mRNA molecules for *PTH1R* and *PTH2R* genes or as percent of the respective levels measured in control groups for *MYOD1*, *MYOGENIN*, and *MYH2* genes. All the information about primers and probes (Integrated DNA Technologies, Coralville, IA, USA) used in the analyses is provided in Table 1.

### 2.5. Cyclic Adenosine Monophosphate (cAMP) Assay

The secondary messenger cAMP was analyzed using the cAMP complete ELISA kit (Abcam, Cambridge, UK, ab133051) to evaluate the functionality of PTH receptors in primary hSC lines and myotubes. For the assay, the hSCs, cultured in growth medium up to 60–70% of confluency, were stimulated with PTH (1-84) at different concentrations, from 10^−6^ to 10^−12^ mol/L, for 30 min at 37 °C. For differentiating experiments, hSCs were previously differentiated in myotubes and then stimulated with PTH (1-84).

Cells treated with 10 µmol/L Forskolin (FSK, Sigma-Aldrich, St. Louis, MO, USA) were used as positive control. The hSCs/myotubes were stimulated independently in quadruplicates for each stimulation in a 24-multiwell culture dish with 250 µL of culture medium containing PTH for 30 min at 37 °C in modified air with 5% CO_2_.

The cell lysates from each well were obtained adding a fresh solution of 150 µL of 0.1 mol/L HCl (Carlo Erba Reagents Srl, Cornaredo, Italy) + 0.5% *v/v* Triton X-100 (Sigma-Aldrich, St. Louis, MO, USA) to each well, and cAMP ELISA assay was performed according to the kit instructions. The absorbance was read using a spectrophotometric microplate reader (TECAN, Infinite 200 PRO, equipped with i-control^TM^ Software, No.: 1.6, Salzburg, Austria). Data were normalized on total protein content and cAMP levels were expressed as pmol/mg of proteins.

### 2.6. Cell Proliferation Assay

Bromodeoxyuridine (BrdU) incorporation assay was performed in hSCs using a commercial ELISA kit (Abcam, Cambridge, UK, ab126556). Briefly, cells were plated in flat-bottomed 96-well microplates at 1 × 10^4^ cells/well. The day after plating, cells were starved for 24 h and then incubated in proliferation medium with different concentrations of PTH (1-84) for 30 min. Cells were then incubated with BrdU 24 h before the end of experiments and kit was performed in accordance with the manufacturer’s instructions. Data were expressed as percent of the respective absorbance measured in control group.

### 2.7. Human MHC Assay

Human MHC was assessed using the ELISA kit (LSBio, Seattle, WA, USA, LS-F31979) to quantify the production of MHC protein in developed myotubes, obtained differentiating hSCs for 6 days in presence, or not, of an initial stimulus of PTH (1-84) at 10^−7^ mol/L for 30 min. Myotubes were detached and pellets resuspended in 180 μL Dulbecco’s phosphate-buffered saline (Sigma-Aldrich, St. Louis, MO, USA). Samples were sonicated followed by 3 cycles of 10 s each (1 impulse/s) and MHC ELISA kit was performed following manufacturer’s instructions. Data were normalized on total protein content and MHC production was expressed as percent of the respective levels measured in control.

### 2.8. Statistical Analysis

Each experiment was performed a minimum of three times. Data are expressed as means ± SD, and statistical significance was determined by Student’s *t*-test or one-way ANOVA analysis with Bonferroni’s multiple comparison test, using GraphPad Prism 9 Software (San Diego, CA, USA). *p* ≤ 0.05 was considered statistically significant.

## 3. Results

### 3.1. Phenotype of Human Satellite Cells in Culture

**Figure 1:**
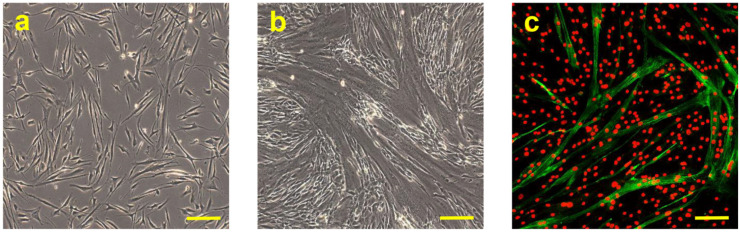
Representative observation of primary culture of hSCs in phase-contrast microscopy, objective 10×, scale bar 200 µm (**a**); representative observation in phase-contrast microscopy of the multinucleate cells after 6 days of myogenic induction, objective 10×, scale bar 200 µm (**b**). Representative fluorescence observation of MHC protein produced by hSCs after 6 days in myogenic induction. MHC, present in myotubes, is stained green (Alexa Fluor 488) and nuclei are stained red (ethidium bromide), objective 10×, scale bar 200 µm (**c**).

The primary cultures of the hSCs were isolated and amplified as reported in the Materials and Methods. The adherent cells showed an elongated morphology with 2–4 cytoplasmic extensions (Figure 1a).

Confluent hSCs, at 70–80% density in the plate, were differentiated toward the myogenic phenotype using an appropriate myogenic differentiation medium for 6 days. During this period, the activated cells align (myoblasts), then subsequently fuse with each other, and finally differentiate into multinucleated myofibers. Phase-contrast microscopy revealed the presence of multinucleated elongated cells, containing from three to more than eight nuclei, related to myotubes (Figure 1b).

After 6 days of myogenic induction, the multinucleated cells exhibited high MHC expression, which is the most important terminal myogenic differentiation marker [27], as confirmed by immunofluorescence microscopy (Figure 1c).

### 3.2. Expression Analysis of PTH1R and PTH2R

**Figure 2:**
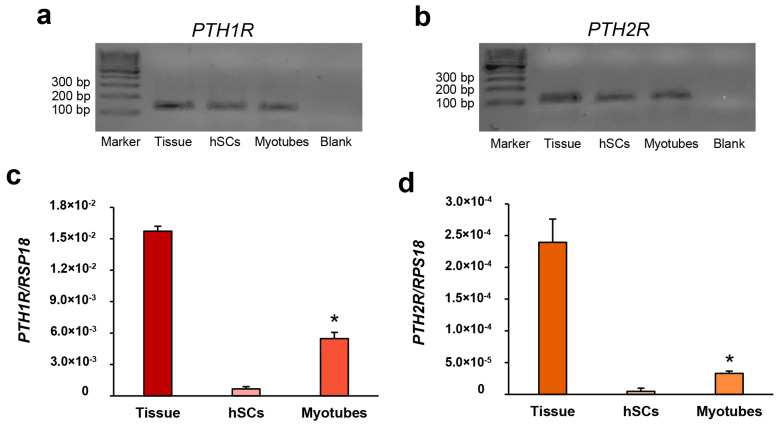
Qualitative PCR analysis has shown the presence of the *PTH1R* (**a**) and *PTH2R* (**b**) mRNA genes in skeletal muscle tissue, hSCs, and differentiated myotubes of the three established hSC lines. Reported blots are representative of the three established cell lines. Marker 100 base pair (bp). Quantitative RealTime-qPCR analysis of the *PTH1R* (**c**) and *PTH2R* (**d**) in skeletal muscle tissue, in hSCs, and in differentiated myotubes. Values are the means ± SD of three cell lines repeated in triplicate and are expressed as the number of mRNA molecules of the gene normalized to the housekeeping gene *RPS18* mRNA. ANOVA analysis with Bonferroni’s multiple comparison test. * *p* ≤ 0.001 compared to hSCs.

Human skeletal muscle tissue, hSCs, and myotubes were analyzed by qPCR in order to evaluate the presence of the *PTH1R* and *PTH2R* mRNAs in our model. As shown in Figure 2a,b, the results of the hormone receptor genes demonstrate evidence of the presence of both mRNAs in the skeletal muscle tissues, in the hSCs, and in the differentiated myotubes, supporting the hypothesis that PTH may be able to interact with skeletal muscle via its receptors.

Moreover, the RealTime-qPCR of the *PTH1R* and *PTH2R* was performed in human skeletal muscle tissue, hSCs, and differentiated myotubes in order to quantify the gene expressions in our cellular models. As reported in Figure 2c,d, the analysis has shown significant increments of *PTH1R* and *PTH2R* expressions in differentiated myotubes with respect to hSCs.

### 3.3. Effect of PTH (1-84) Treatment on cAMP Levels in Proliferated hSCs and Differentiated Myotubes

The action of PTH through its receptors is mainly mediated by the activation of cAMP upon a G-protein-mediated cascade of events [28].

**Figure 3:**
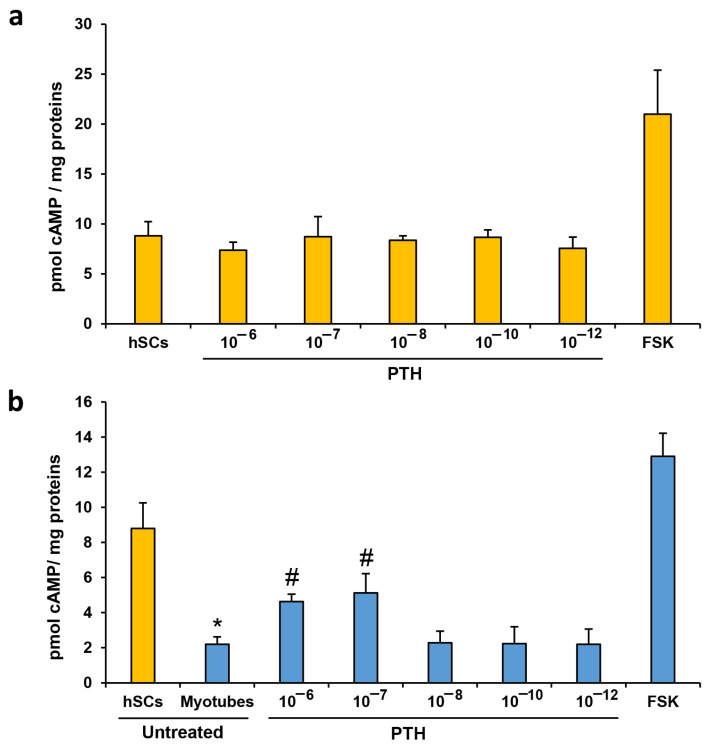
cAMP levels in proliferating hSCs (**a**) and in differentiated myotubes (**b**) treated for 30 min with different concentrations of PTH (1-84), ranging from 10^−6^ to 10^−12^ mol/L and with FSK 10 µmol/L used as positive control. The cAMP values, normalized on total protein content and expressed as pmol/mg of proteins, are the mean ± SD of three cell lines repeated in quadruplicate. ANOVA analysis with Bonferroni’s multiple comparison test. # *p* ≤ 0.05 vs. untreated myotubes, * *p* ≤ 0.001 vs. untreated hSCs.

As shown in Figure 3a, the 30-min PTH (1-84) treatment at different concentrations did not affect the cAMP levels in the proliferating hSCs. On the other hand, the same treatments performed on the differentiated myotubes have shown significant increases in the cAMP accumulation in cells treated with 10^−7^ and 10^−6^ mol/L with respect to the untreated myotube controls, with an increment of +113% and +80%, respectively (Figure 3b). Moreover, we found lower baseline levels of cAMP in the differentiated myotubes (−75%) with respect to the value in untreated hSCs.

### 3.4. Effect of PTH (1-84) Treatment on hSC Proliferation Process

**Figure 4:**
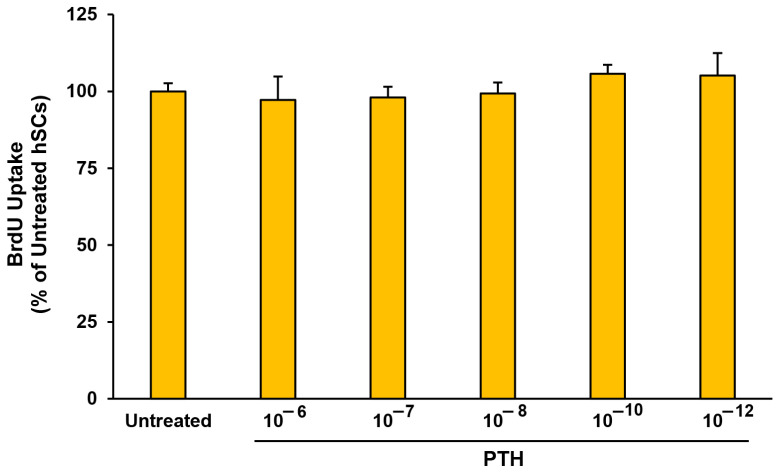
Effects of 30-min PTH (1-84) treatment at different concentrations on hSC proliferation process assessed by BrdU incorporation proliferation assay. The values, expressed as percentage of untreated hSCs (control), are the mean ± SD of three cells lines repeated in quadruplicate. ANOVA analysis with Bonferroni’s multiple comparison test.

The analysis has shown no significant differences between the hSCs stimulated with different concentrations of PTH (1-84), from 10^−6^ to 10^−12^ mol/L, for 30 min and the untreated control, indicating that the hormone does not affect the hSCs’ proliferation under the described experimental conditions (Figure 4).

### 3.5. Effect of PTH (1-84) Treatment on Gene Expression during hSC Differentiation

**Figure 5:**
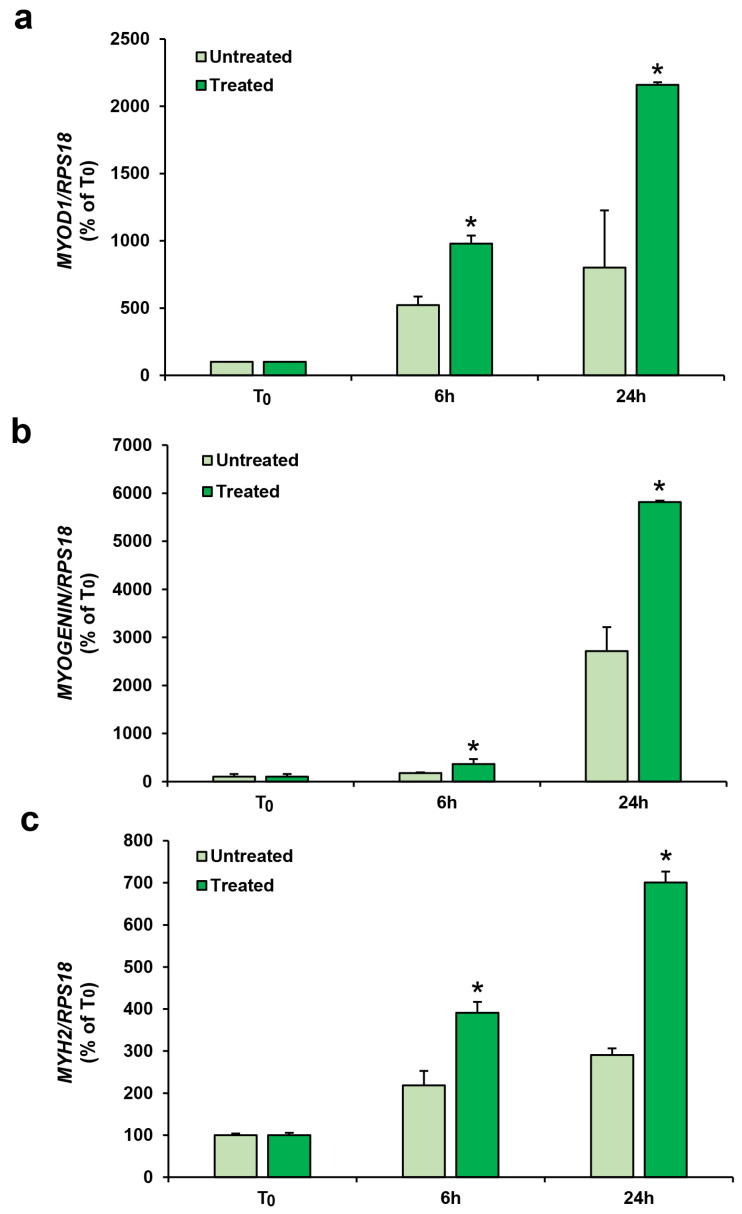
RealTime-qPCR gene expression analysis of *MYOD1* (**a**), *MYOGENIN* (**b**), and *MYH2* (**c**) detected during the early stages of hSC differentiation, treated or not with 10^−7^ mol/L of PTH (1-84) for 30 min. Analysis was performed on cells in growth medium (T_0_) and after 6 and 24 h of myogenic differentiation. Values are expressed as percentage of the T_0_ and are the mean ± SD of three cell lines repeated in triplicate, normalized to the housekeeping gene RPS18. ANOVA analysis with Bonferroni’s multiple comparison test. * *p* ≤ 0.001 versus related untreated group.

The molecular analysis during the early stages of the differentiation showed significant increases in the expression of the myogenic differentiation genes after the 30-min treatment with 10^−7^ mol/L PTH (1-84) versus the untreated control groups, indicating its direct effect on myogenesis (Figure 5).

In particular, the *MYOD1* expression was increased by +87.68% 6 h after and +169.5% 24 h after the PTH (1-84) treatment with respect to the related untreated groups (Figure 5a). The *MYOGENIN* expression was increased +107.3% 6 h after and +112.7% 24 h after the treatment with PTH (1-84) with respect to the related untreated groups (Figure 5b). The *MYH2* expression was increased +79% 6 h after and +141% 24 h after the PTH (1-84) treatment with respect to the related untreated groups (Figure 5c).

### 3.6. Effect of PTH (1-84) Treatment on MHC Protein Levels

**Figure 6:**
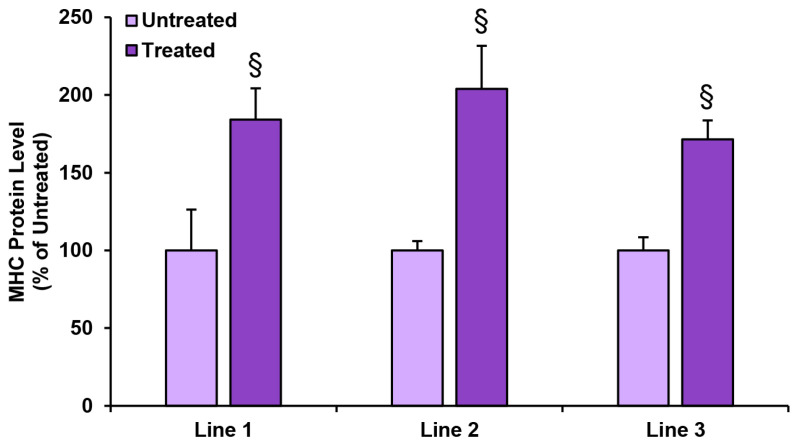
Levels of MHC protein detected in hSCs differentiated in myotubes, treated or not with 10^−7^ mol/L PTH (1-84) for 30 min, after 6 days of myogenic differentiation. Values of MHC protein, normalized on total protein content and expressed as percentage of untreated myotubes (control), are the mean ± SD of three experiments repeated in quadruplicate. Student’s *t*-test analysis. § *p* ≤ 0.01 compared to related untreated line.

In all three established cell lines, the data showed significant increases in MHC production in the groups treated with a single impulse of the 30 min PTH (1-84) treatment at the concentration of 10^−7^ mol/L with respect to the control groups, supporting a direct effect of the hormone on skeletal muscle differentiation. Specifically, the quantification of the MHC protein in the myotubes treated with PTH (1-84) resulted in significant gains of +84.2%, +103.9%, and +71.5% of the MHC protein, compared to the related untreated line (Figure 6).

In Table 2, the individual data points of the MHC protein quantification for each cell line differentiated in the myotubes are reported.

**Figure 7:**
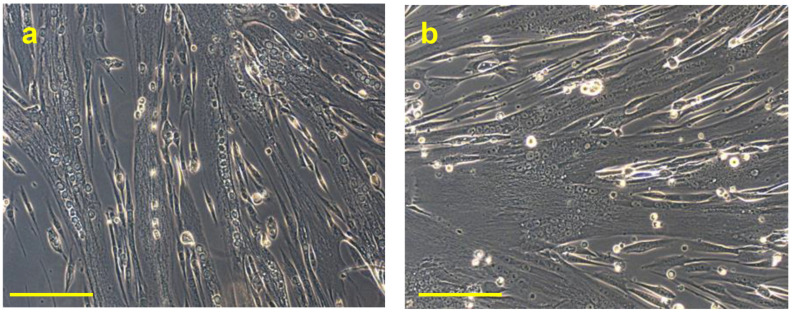
Representative observations acquired in phase-contrast microscopy of the myotubes, obtained by hSCs untreated (**a**) or treated (**b**) with 10^−7^ mol/L PTH (1-84) for 30 min, after 6 days of myogenic differentiation. Objective 20×, scale bar 50 µm.

Figure 7 reports the images acquired by phase-contrast microscopy of the myotubes obtained after 6 days of myogenic differentiation, obtained by hSCs untreated (a) or treated (b) with 10^−7^ mol/L PTH (1-84) for 30 min. These observations clearly demonstrate a consistent size of diameter and the increase in the number of nuclei inside the myotubes treated with the hormone, compared to the untreated.

## 4. Discussion

The objective of the present research was to evaluate the effect of the 30-min exposure time to the full-length PTH (1-84) on the proliferation and the myogenesis of skeletal muscle SCs isolated from human biopsies.

As supported by the literature [10,13], we have shown the presence of the *PTHR1* and *PTHR2* mRNAs in human skeletal muscle tissue and in our cellular models (Figure 2). Moreover, the quantification of the *PTHR1* and *PTH2R* has shown increased expression of the genes during myogenesis. The data on the *PTH1R* are in agreement with our previous research where we highlighted the importance that this receptor has during hSCs’ differentiation and myotube maturation [25].

To confirm the interaction of hormone–receptors, we assayed the cAMP levels, treating hSCs and myotubes with 30-min stimuli of PTH (1-84) at different concentrations. For the first time, the results show evidence that 30-min PTH treatments do not affect intracellular cAMP production in proliferating hSCs at any of the tested concentrations, whereas in myotubes only 10^−6^ and 10^−7^ mol/L concentrations are able to trigger significant increases in the cAMP levels (Figure 3). The data obtained, with higher concentrations in the myotubes, are in agreement with the previous research in which the same dosages were responsible for the activation of the cAMP pathway in UMR106 osteogenic sarcoma cells [28]. The fact that PTH apparently affects only cAMP levels in myotubes can be explained by considering that the basal levels of cAMP in hSCs are high and become low after hSC differentiation in myotubes (Figure 3b). These data are in line with the literature, as intracellular cAMP normally declines after myoblast fusion [29]. In fact, cAMP signaling is dynamically regulated during muscle development and regeneration, and cAMP levels decrease in muscle precursor cell differentiation, migration, and fusion, which are all cellular events required for the efficient regeneration of adult skeletal muscle [30].

We therefore believe that we did not detect any cAMP variations in the hSCs because the cells already have high cAMP levels, and the 30-min stimuli with the hormone are not able to further raise the baseline levels. This does not mean that PTH does not interact with its receptors in hSCs but perhaps that this interaction is not relevant in terms of cAMP production. On the other hand, variations in the intracellular cAMP levels are detected in myotubes, because these levels are physiologically declined and, thus, 30-min stimuli with higher PTH (1-84) concentrations are able to enhance the cAMP production.

Some works in the literature report that transient treatment with catecholamines or prostaglandine E_1_, which stimulate intracellular cAMP production, enhances the fusion of primary chick myoblasts [31,32]. However, intracellular cAMP normally declines after myoblast fusion and sustained cAMP signaling potently inhibits myogenic differentiation, in part by inhibiting muscle-specific transcription by silencing the activity of the myogenic family of regulatory factors, which includes MYOD1, MYOGENIN, myogenic factor 5 (MYF5), and myogenic regulatory factor 4 (MRF4) [33,34]. Therefore, future therapeutic attempts to stimulate myoblast fusion must be designed with caution in order to allow a dynamic cAMP regulation [29].

Because a wide range of intracellular processes are influenced by cAMP, our study has evaluated whether 30-min PTH (1-84) treatments can influence the proliferation process of hSCs. The data are evidence that proliferation is not affected by a 30-min stimulation with the hormone at different concentrations (Figure 4). Conversely, a previous study on the murine myoblast C_2_C_12_ line treated for 72 h with several PTH (1-34) concentrations, ranging from 10^−8^ to 10^−11^ mol/L, highlighted that the hormone is able to induce C_2_C_12_ proliferation after in vitro administration with all the treatments tested, leading us to assume that an extended length of treatments may influence the proliferation of hSCs, as reported in the C_2_C_12_ cells [21].

Numerous experimental conditions are used by varying the length and concentration of the stimulus with PTH. In this regard, studies in the literature have shown that exposure to PTH (1-34) for the first 6 h of repeated 48 h cycles, at the concentrations of 10^−8^ or 10^−7^ mol/L, were able to promote the proliferation of rat bone mesenchymal stromal cells and human periodontal stem cells, respectively [35,36]. However, a study reports that different PTH exposure modalities (stimulations at 10^−10^ mol/L PTH (1-34), continuatively or in a pulsatile fashion for 6 h every 48 h, for an experimental time of 14 days) were not found to influence the proliferation process of rat bone marrow stromal cells [37]. Therefore, considering these studies, the cellular proliferative effect is likely related to exposure times, modalities, and concentrations, making PTH studies challenging.

Interestingly, for the first time in human skeletal muscle cells, we show (Figure 5) that a single short impulse of 30-min PTH (1-84), at the higher concentration tested (10^−7^ mol/L), is able to affect the early stage of hSC differentiation, positively increasing the in vitro gene expression of myogenic markers (*MYOD1*, *MYOGENIN*, and *MYH2*). Moreover, effective increases in the MHC protein assayed in the myotubes, obtained with the 30-min stimuli of PTH (1-84) and after 6 days of hSC myogenic differentiation, confirm the remarkable effect of PTH (1-84) on human myogenesis (Figure 6). Our data for human muscle cells are in agreement with the PTH (1-34)-induced murine C_2_C_12_ muscle cell differentiation, detected by Western blot [21].

The role of PTH in the induction of cell differentiation is shown in other tissues. In particular, the intermittent administration of PTH (1-34) increases bone mass by promoting the osteogenesis of human adipose-derived mesenchymal stem cells or periodontal ligament stem cells [37,38,39]. Moreover, it has been shown that PTH (1-34) promotes the chondrogenic differentiation of bone marrow mesenchymal stem cells under specific experimental conditions [40].

In summary, our findings show that a single impulse of 30 min of PTH (1-84), albeit not appearing to be able to affect the in vitro proliferation process of hSCs, results in being sufficient to promote the myogenic differentiation process.

Although the data are encouraging, the present study has some limitations. First, we used a small number of cellular lines established by three biopsies derived from different muscle groups, with different muscle fiber compositions and types. Second, the biopsies were obtained by healthy female subjects with different ages, ranging from 29 to 52 years old. Third, the circulating level of PTH in these volunteers was not known. However, despite such variables, the presented results clearly show that 30-min PTH treatment can play an important role in the development and regeneration of human skeletal muscle. In particular, although the absolute values of the MHC protein levels (Table 2) result in being different for each line, assuming an individual baseline variability and speculating that the myogenic potential may differ for each patient, the assayed MHC levels increased in all three cell lines after 30-min PTH (1-84) exposure.

Further studies are needed to examine the effect of PTH (1-84) using different exposure times and different concentrations of PTH.

## 5. Conclusions

The present study demonstrates for the first time the potential effects of 30-min PTH (1-84) treatment on hSCs isolated from patient biopsies, confirming that PTH acts on the regulation of non-traditional target organs, such as skeletal muscle tissue and the myogenesis of hSCs, opening new avenues of investigation on the role of the hormone in muscle pathophysiology. It is conceivable that, as it is already for bone regeneration, PTH may be a promising and novel therapeutic strategy for regulating different processes of skeletal muscle in those conditions in which there is muscle dysfunction due to hypoparathyroidism.

## Figures and Tables

**Table 1:** List of the human gene specific primers and/or specific TaqMan probes used in the analysis.

Gene	Primer and Probe Sequences	T_m_ (°C)	Amplicon Size (bp)
*ACTB*	Forward 5′-AGCCTCGCCTTTGCCGA-3′ Reverse 5′-CTGGTGCCTGGGGCG-3′	60 °C	174
*PTH1R*	Forward 5′-GGGAAGCCCAGGAAAGATAAG-3′ Reverse 5′-CACAGGATGTGGTCCCATT-3′ Probe 6-FAM/TGCCTCCTT/ZEN/GTCCTCCTCAGACTC/3IABkFQ	58 °C	125
*PTH2R*	Forward 5′-AAACATGGGCCAATTATTCAGAC-3′ Reverse 5′-AAAGAGATGGAGTAGCCAACG-3′ Probe 6-FAM/CCTTCGCTT/Zen/TCTGCAGCCAGATATCA/3IABkFQ	58 °C	118
*MYOD1*	Forward 5′-GACGTGCCTTCTGAGTCG-3′ Reverse 5′-CTCAGAGCACCTGGTATATCG-3′ Probe 6-FAM/CGCTGCTCT/Zen/CTCCCTCGCTG/3IABkFQ	55 °C	148
*MYOGENIN*	Forward 5′-AGCGAATGCAGCTCTCAC-3′ Reverse 5′-TGTGATGCTGTCCACGATG-3′ Probe 6-FAM/TGACCCTAC/Zen/AGATGCCCACAACC/3IABkFQ	55 °C	150
*MYH2*	Forward 5′-CCAGACTGTGTCTGCTCTCTTCAG-3′ Reverse 5′-CAGGACAAGCTCATGCTCCAT -3′ Probe 6-FAM/CAAGTCTTC/Zen/CCCATGAACCCTCCC/3IABkFQ	60 °C	139
*RPS18*	Forward 5′- CTGCTGCTTTGAGTGTGTGG-3′ Reverse 5′- CTTGGCAATGCAGGAGGTGT-3′ Probe 6-FAM/TTCAGGGAT/ZEN/CACTAGAGACATGGCTGC/3IABkFQ	60 °C	132

TaqMan probes have F as reporter fluorochrome [6-carboxyfuorescein (6-FAM)] and ZEN as quencher. Fluorochrome (Iowa Black FQ); bp, base pairs of amplicon size; Tm, melting temperature (°C).

**Table 2:** Individual data points of MHC protein quantification in each hSC line differentiated in myotubes.

Line 1		Line 2		Line 3	
Control	PTH	Control	PTH	Control	PTH
1.36	2.10	1.55	2.87	3.25	5.97
0.96	2.11	1.76	3.02	3.52	5.89
1.04	2.50	1.62	3.51	3.90	6.23
1.69	2.59	1.56	3.84	3.88	6.86

MHC protein expression was detected in hSCs differentiated in myotubes, treated or not with 10^−7^ mol/L PTH (1-84) for 30 min, after 6 days of myogenic differentiation and data are expressed as ng/mg of proteins.

## Data Availability

The data sets analyzed in the current study are not publicly accessible, but they are available from the corresponding author upon reasonable request.

## References

[1] Cianferotti L., Romagnoli C., Brandi M.L., Ulloa-Aguirre A., Tao Y.-X. (2021). Chapter 9—Sensing Calcium Levels: The Biology of the Parathyroid Cells. Cellular Endocrinology in Health and Disease.

[2] Brown E.M. (2000). Calcium Receptor and Regulation of Parathyroid Hormone Secretion. Rev. Endocr. Metab. Disord..

[3] Bergwitz C., Jüppner H. (2010). Regulation of Phosphate Homeostasis by PTH, Vitamin D, and FGF23. Annu. Rev. Med..

[4] Gardella T.J., Vilardaga J.-P. (2015). International Union of Basic and Clinical Pharmacology. XCIII. The Parathyroid Hormone Receptors--Family B G Protein-Coupled Receptors. Pharmacol. Rev..

[5] Sutkeviciute I., Clark L.J., White A.D., Gardella T.J., Vilardaga J.P. (2019). PTH/PTHrP receptor signaling, allostery, and structure. Trends Endocrinol. Metab..

[6] Usdin T.B., Gruber C., Bonner T.I. (1995). Identification and functional expression of a receptor selectively recognizing parathyroid hormone, the PTH2 receptor. J. Biol. Chem..

[7] Usdin T.B., TI Bonner T.I., Harta G., Mezey E. (1996). Distribution of parathyroid hormone-2 receptor messenger ribonucleic acid in rat. Endocrinology.

[8] Hoare S.R., Usdin T.B. (2001). Molecular Mechanisms of Ligand Recognition by Parathyroid Hormone 1 (PTH1) and PTH2 Receptors. Curr. Pharm. Des..

[9] Hoare S.R., Bonner T.I., Usdin T.B. (1999). Comparison of rat and human parathyroid hormone 2 (PTH2) receptor activation: PTH is a low potency partial agonist at the rat PTH2 receptor. Endocrinology.

[10] Tian J., Smogorzewski M., Kedes L., Massry S.G. (1993). Parathyroid Hormone-Parathyroid Hormone Related Protein Receptor Messenger RNA Is Present in Many Tissues besides the Kidney. Am. J. Nephrol..

[11] Kousteni S., Bilezikian J.P. (2008). The Cell Biology of Parathyroid Hormone in Osteoblasts. Curr. Osteoporos. Rep..

[12] Silva B.C., Costa A.G., Cusano N.E., Kousteni S., Bilezikian J.P. (2011). Catabolic and Anabolic Actions of Parathyroid Hormone on the Skeleton. J. Endocrinol. Investig..

[13] Ureña P., Kong X.F., Abou-Samra A.B., Jüppner H., Kronenberg H.M., Potts J.T., Segre G.V. (1993). Parathyroid Hormone (PTH)/PTH-Related Peptide Receptor Messenger Ribonucleic Acids Are Widely Distributed in Rat Tissues. Endocrinology.

[14] Reppe S., Stilgren L., Abrahamsen B., Olstad O.K., Cero F., Brixen K., Nissen-Meyer L.S., Gautvik K.M. (2007). Abnormal Muscle and Hematopoietic Gene Expression May Be Important for Clinical Morbidity in Primary Hyperparathyroidism. Am. J. Physiol. Endocrinol. Metab..

[15] Garber A.J. (1983). Effects of Parathyroid Hormone on Skeletal Muscle Protein and Amino Acid Metabolism in the Rat. J. Clin. Investig..

[16] Baczynski R., Massry S.G., Magott M., el-Belbessi S., Kohan R., Brautbar N. (1985). Effect of Parathyroid Hormone on Energy Metabolism of Skeletal Muscle. Kidney Int..

[17] Romagnoli C., Brandi M.L. (2021). Muscle Physiopathology in Parathyroid Hormone Disorders. Front. Med..

[18] Cusano N.E., Rubin M.R., McMahon D.J., Irani D., Tulley A., Sliney J., Bilezikian J.P. (2013). The Effect of PTH (1-84) on Quality of Life in Hypoparathyroidism. J. Clin. Endocrinol. Metab..

[19] Tabacco G., Tay Y.-K.D., Cusano N.E., Williams J., Omeragic B., Majeed R., Almonte M.G., Rubin M.R., Bilezikian J.P. (2019). Quality of Life in Hypoparathyroidism Improves With RhPTH(1-84) Throughout 8 Years of Therapy. J. Clin. Endocrinol. Metab..

[20] Sato C., Miyakoshi N., Kasukawa Y., Nozaka K., Tsuchie H., Nagahata I., Yuasa Y., Abe K., Saito H., Shoji R. (2021). Teriparatide and Exercise Improve Bone, Skeletal Muscle, and Fat Parameters in Ovariectomized and Tail-Suspended Rats. J. Bone Miner. Metab..

[21] Fujimaki T., Ando T., Hata T., Takayama Y., Ohba T., Ichikawa J., Takiyama Y., Tatsuno R., Koyama K., Haro H. (2021). Exogenous Parathyroid Hormone Attenuates Ovariectomy-Induced Skeletal Muscle Weakness in Vivo. Bone.

[22] Brent M.B., Brüel A., Thomsen J.S. (2018). PTH (1-34) and Growth Hormone in Prevention of Disuse Osteopenia and Sarcopenia in Rats. Bone.

[23] Yoon S.-H., Grynpas M., Mitchell J. (2019). Intermittent PTH Treatment Improves Bone and Muscle in Glucocorticoid Treated Mdx Mice: A Model of Duchenne Muscular Dystrophy. Bone.

[24] Kimura S., Yoshioka K. (2014). Parathyroid Hormone and Parathyroid Hormone Type-1 Receptor Accelerate Myocyte Differentiation. Sci. Rep..

[25] Romagnoli C., Zonefrati R., Sharma P., Innocenti M., Cianferotti L., Brandi M.L. (2020). Characterization of Skeletal Muscle Endocrine Control in an In Vitro Model of Myogenesis. Calcif. Tissue Int..

[26] Romagnoli C., Sharma P., Zonefrati R., Palmini G., Lucattelli E., Ward D.T., Ellinger I., Innocenti M., Brandi M.L. (2021). Study of the Expression and Function of Calcium-Sensing Receptor in Human Skeletal Muscle. Int. J. Mol. Sci..

[27] Schiaffino S., Reggiani C. (2011). Fiber Types in Mammalian Skeletal Muscles. Physiol. Rev..

[28] Martin T.J. (2021). PTH1R Actions on Bone Using the CAMP/Protein Kinase A Pathway. Front. Endocrinol..

[29] Berdeaux R., Stewart R. (2012). CAMP Signaling in Skeletal Muscle Adaptation: Hypertrophy, Metabolism, and Regeneration. Am. J. Physiol. Endocrinol. Metab..

[30] Wahrmann J.P., Luzzati D., Winand R. (1973). Changes in adenyl cyclase specific activity during differentiation on an established myogenic cell line. Biochem. Biophys. Res. Commun..

[31] Curtis D.H., Zalin R.J. (1981). Regulation of muscle differentiation: Stimulation of myoblast fusion in vitro by catecholamines. Science.

[32] Zalin R.J., Leaver R. (1975). The effect of a transient increase in intracellular cyclic AMP upon muscle cell fusion. FEBS Lett..

[33] Li L., Heller-Harrison R., Czech M., Olson E.N. (1992). Cyclic AMP-dependent protein kinase inhibits the activity of myogenic helix-loop-helix proteins. Mol. Cell. Biol..

[34] Salminen A., Braun T., Buchberger A., Jürs S., Winter B., Arnold H.H. (1991). Transcription of the muscle regulatory gene Myf4 is regulated by serum components, peptide growth factors and signaling pathways involving G proteins. J. Cell Biol..

[35] Chen B., Lin T., Yang X., Li Y., Xie D., Cui H. (2016). Intermittent Parathyroid Hormone (1-34) Application Regulates cAMP-Response Element Binding Protein Activity to Promote the Proliferation and Osteogenic Differentiation of Bone Mesenchymal Stromal Cells, via the CAMP/PKA Signaling Pathway. Exp. Ther. Med..

[36] Du L., Feng R., Ge S. (2016). PTH/SDF-1α Cotherapy Promotes Proliferation, Migration and Osteogenic Differentiation of Human Periodontal Ligament Stem Cells. Cell Prolif..

[37] Yang C., Frei H., Burt H.M., Rossi F. (2009). Effects of Continuous and Pulsatile PTH Treatments on Rat Bone Marrow Stromal Cells. Biochem. Biophys. Res. Commun..

[38] Kuo S.-W., Rimando M.G., Liu Y.-S., Lee O.K. (2017). Intermittent Administration of Parathyroid Hormone 1-34 Enhances Osteogenesis of Human Mesenchymal Stem Cells by Regulating Protein Kinase Cδ. Int. J. Mol. Sci..

[39] Wang X., Wang Y., Dai X., Chen T., Yang F., Dai S., Ou Q., Wang Y., Lin X. (2016). Effects of Intermittent Administration of Parathyroid Hormone (1-34) on Bone Differentiation in Stromal Precursor Antigen-1 Positive Human Periodontal Ligament Stem Cells. Stem Cells Int..

[40] Zhang Y., Kumagai K., Saito T. (2014). Effect of Parathyroid Hormone on Early Chondrogenic Differentiation from Mesenchymal Stem Cells. J. Orthop. Surg. Res..

